# Sensitive detection of *in vivo* traumatic brain injury foci using magnetic particle imaging

**DOI:** 10.1039/d6nr02497a

**Published:** 2026-09-24

**Authors:** Hayden J. Good, Mohammad Tabatabaei, Eiko Alzamora, Neel Eswaran, Sk Rajab Ali, Ambar C. Velazquez-Albino, Zachary D. Simon, Marcelo Febo, Lakiesha N. Williams, Carlos M. Rinaldi-Ramos

**Affiliations:** a Chemical Engineering, University of Florida 1075 Center Dr Gainesville FL 32611 USA carlos.rinaldi@ufl.edu; b J. Crayton Pruitt Department of Biomedical Engineering, University of Florida 1275 Center Dr Gainesville FL 32611 USA LWilliams@bme.ufl.edu; c Department of Materials Science & Engineering, University of Florida 1149 S Newell Dr Gainesville FL 32611 USA; d Department of Psychiatry, McKnight Brain Institute, University of Florida 1149 S. Newell Dr Gainesville FL 32611 USA

## Abstract

Diagnosing traumatic brain injury (TBI) remains a major clinical challenge, particularly for mild to moderate cases, due to the limited sensitivity of conventional imaging modalities like magnetic resonance imaging (MRI) and computed tomography (CT) in detecting subtle cerebrovascular damage early after injury occurs. Magnetic particle imaging (MPI) is a novel modality that offers high sensitivity, linear quantification, and unambiguous detection of superparamagnetic tracers such as iron oxide nanoparticles (IONPs). This study evaluates the capacity of MPI to detect and visualize cerebral bleeding resulting from TBI. A controlled cortical impact (CCI) closed-head TBI model was implemented using a calibrated electromagnetic impactor to induce moderate brain injury in C57BL/6 mice. IONPs were administered intravenously after impact, to mimic potential clinical use. CT-registered MPI images revealed strong, localized signals in the impact region of injured animals at early time points, while sham animals showed no residual signal in the brain. This localized signal decayed over time and eventually approached noise levels after 7 days post-injury. A direct comparison between MPI and 
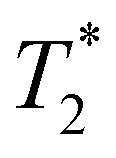
-weighted MRI demonstrated that only MPI reliably differentiates the early injury foci at 24 hours. These results highlight the sensitivity and specificity of MPI as a potential diagnostic tool for detecting vascular disruption in TBI, offering a promising minimally invasive approach for early injury assessment. Additionally, these results demonstrate nanoparticle accumulation in sites of moderate TBI, highlighting potential theranostic applications of MPI to develop drug carriers suitable for treatment of TBI.

## Abstract 2

Diagnosing traumatic brain injury (TBI), especially mild to moderate cases, remains a major clinical challenge because conventional imaging modalities like MRI and CT often lack sensitivity to detect subtle cerebrovascular damage early after injury.

## Graphical abstract

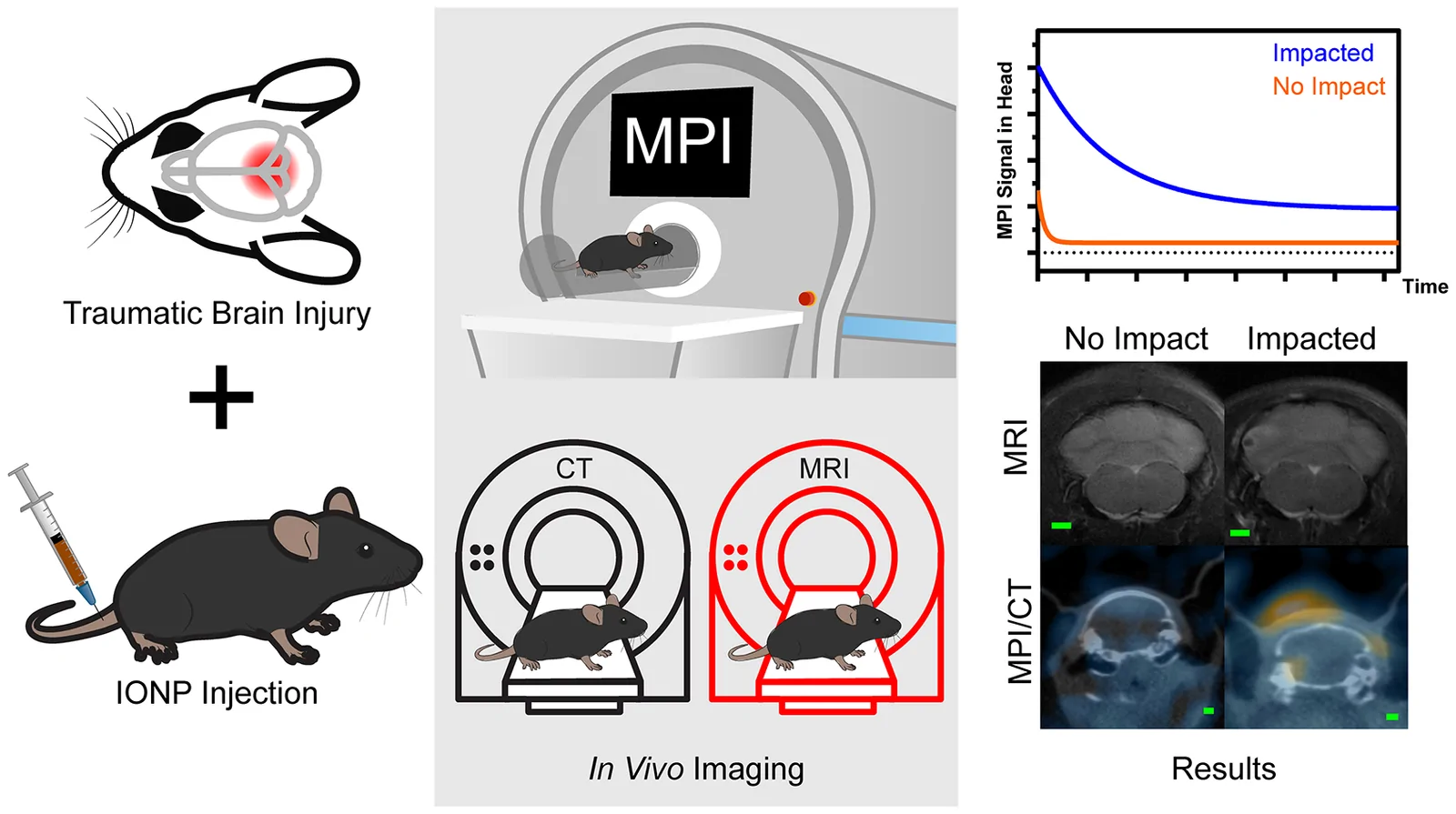

## Introduction

Traumatic brain injury (TBI) is a significant cause of mortality and long-term disability worldwide, with millions of emergency room visits reported annually in the United States alone.^1^ TBI occurs when an external force causes damage to the brain, leading to acute consequences such as intracranial bleeding, edema, blood–brain barrier (BBB) disruption, and chronic outcomes, including neuroinflammation, gliosis, and cognitive impairment.^2,3^ Despite its prevalence, the intricacies of TBI pathophysiology, particularly at the microvascular level, remain poorly understood, hampered significantly by limitations in current diagnostic imaging technologies. Disruption of the BBB is a hallmark of TBI pathology that is classified by altered permeability and the infiltration of immune cells such as microglia and astrocytes.^4^ This imbalance in the BBB contributes to secondary injury mechanisms in the brain.^5^ Secondary effects of TBI may persist for years after the initial brain injury. The secondary sequelae exacerbate neuroinflammation and lead to further neurodegeneration and neurological deficits. The ability to monitor and potentially treat the aggressive neuroimmune response that occurs in the brain hours post-TBI may mitigate the progression of secondary brain injury.^6^

Computed tomography (CT) and magnetic resonance imaging (MRI) are the current clinical gold standard brain imaging modalities for assessing TBI. They are effective at evaluating large-scale defects such as epidural and subdural bleeding, contusion, and skull fracture. CT remains a critical first pass exam, as it can quickly detect life-threatening conditions that require immediate attention, such as acute severe TBI and stroke.^7,8^ MRI holds potential to inform on the pathophysiology of TBI by suggesting the presence of early cytotoxic and vasogenic edema, but it is not sufficient alone for diagnosis.^9,10^ These modalities offer limited sensitivity in detecting microscopic bleeding and subtle injuries during the early stages of TBI, a critical time in the injury process.^11^ This limitation has impeded efforts to elucidate the pathophysiological role of cerebral microbleeds and their relationship to other aspects of TBI pathology, such as diffuse axonal injury and long-term neurodegeneration.^12^ Furthermore, mild and moderate TBI are difficult to diagnose using MRI due to issues with ambiguous signal and low signal-to-noise ratio (SNR).^10,13^ For instance, molecular species may spill into surrounding tissue following a TBI event, degrading SNR, reducing specificity, and undermining the ability to determine vascular and tissue-type contributions to the measured MRI signals. Real-time tracking using molecular tracers could offer essential insights into the extent and temporal evolution of vascular injury.

Magnetic particle imaging (MPI) is a novel imaging modality where an applied magnetic field selectively excites then captures signal from superparamagnetic material, often in the form of iron oxide nanoparticles (IONPs).^14,15^ This modality has several advantages that make it attractive for TBI imaging, including the unambiguous signal arising solely from engineered exogenous biologically inert nanoparticle tracers, its high sensitivity, linearly quantitative signal, adequate resolution, and the ability to measure signal over long periods.^16,17^ Further, MPI does not rely on ionizing radiation, and the IONPs tracers are shelf-stable for prolonged periods, avoiding potential radiological safety and logistical complications associated with positron emission tomography.^18^ Prior studies have demonstrated the potential of MPI in several biomedical applications, including immune cell tracking,^19–24^ image-guided hyperthermia,^25–27^ vascular imaging,^28–30^ and blood pool imaging.^31–33^

The potential of MPI to diagnose and study TBI was suggested in a proof-of-principle study by Orendorff *et al.*,^32^ who evaluated the accumulation of intravenously administered SPIONs at a site of brain injury and their subsequent clearance and redistribution to draining lymph nodes. While promising, some limitations of this work included the use of a poorly characterized injury model, administration of IONPs prior to injury, reporting observations based on a single animal per condition, and a lack of comparisons to other assessments of TBI. Here, we provide more rigorous evidence of the potential of MPI to detect TBI when IONPs are administered post-injury, and by comparing MPI and MRI measures of TBI in a validated closed cortical injury model.^34^ These studies demonstrate that MPI can monitor IONP accumulation and clearance from the region of the head subjected to TBI.

## Materials and methods

### Synthesis of iron oleate precursor

Iron oleate was prepared according to published work with some modifications.^35^ Iron(iii) acetylacetonate (11.19 g, 31.68 mmol) was combined with oleic acid (44.74 g, 158.40 mmol) in a 1 : 5 molar ratio inside a 250 mL 3-neck round-bottom flask. The reactor was placed in a heating mantle, with a condenser, thermocouple, and overhead stirrer in each neck. The condenser was connected to a chiller set to 12 °C and attached to the right neck of the reactor. The overhead stirrer, set up in the middle neck, was set to a rate of 350 rpm. The thermocouple and gas-flow needle were held by a septum in the left neck, with the argon flow set to 100 sccm. Once the equipment was set up, the temperature controller was set to 325 °C. Once the reaction reached 300 °C, aliquots were taken with a syringe and stainless-steel needle through the septum in the left neck. FTIR was used to scan aliquots in real time and determine the reaction end point, controlling the percentage of free oleic acid left in the oleate. Once the free oleic acid present in the precursor mixture was estimated to be close to 35%, the reactor was removed from the heating mantle and allowed to cool to room temperature before leaving it under vacuum overnight, prior to iron oxide nanoparticle synthesis the next day.

### Synthesis of iron oxide nanoparticles

First, 13 mL of oleyl alcohol (34.98 mmol based on 85% purity) were added to a 100 mL three-neck-flask, placed in a heating mantle, and mixed using a borosilicate glass coated magnetic stir bar and stir plate. The reactor's left neck was connected to a Schlenk line, and the middle neck had a rubber septum with a thermocouple. A glass stopper was added to the right neck of the reactor before starting a vacuum treatment for 1 hour while heating to 140 °C. After the vacuum treatment, the reactor was purged with argon. The flow rate of argon through the reactor was set to 100 sccm using a mass flow controller, and the glass stopper in the right neck of the reactor was removed. Then, the reactor was wrapped with quartz wool insulation before heating to 320 °C. After reaching the reaction temperature in the reactor, 10 mL of the iron oleate was dripped from the middle neck of the reactor at a controlled flow rate of 0.16 mL min^−1^ using a syringe pump.

### Iron quantification

A 1,10-phenanthroline colorimetric assay was used to quantify the iron concentration of nanoparticle solutions,^36^ described previously.^37^ Briefly, 10 µL of nanoparticle solution was digested in 1 mL of nitric acid overnight. An aliquot was taken of the digested solution and added to deionized water and hydroxylamine hydrochloride for 1 h. Then, sodium acetate and 1,10-phenanthroline monohydrate were added. An aliquot of the resulting solution was placed in a 96-well polystyrene plate, and absorbance was measured at 508 nm in a SpectraMax M5 microplate reader. The concentration of each sample run in triplicate was determined by relating the absorbance to a calibration curve prepared from an iron standard solution.

### Nanoparticle purification

Nanoparticles were purified using hexane and ethanol solvent–antisolvent washes with circular magnetic Halbach arrays to remove reaction byproducts and any remaining reagents. Synthesized nanoparticles were well homogenized after storage, and 1 mL of the reaction mixture was added to a 15 mL centrifuge tube. This was followed by the addition of 0.3 mL of hexane, vortexing, and addition of 1 mL of ethanol. This solution was lightly mixed before inserting into a circular Halbach array for 10 minutes. The supernatant was discarded by decanting, keeping the tube in the Halbach array, and nanoparticles were resuspended in 0.75 mL of hexane. After mixing and adding 1 µL of oleic acid, cup horn sonication was used for 1 minute at 80% amplitude. Again, 1 mL of ethanol was added to the nanoparticle solution, lightly mixed, and inserted into the Halbach array for 10 minutes. The supernatant was discarded, and the process of adding hexane and oleic acid, then ethanol, and insertion into the Halbach array was repeated two more times. After the final supernatant was discarded, nanoparticles were resuspended in 1 mL toluene. Aliquots were taken to perform iron quantification prior to the ligand exchange process.

### Polymer coating of IONPs

Monomethyl polyethylene glycol (mPEG-5000) from BioPharma was functionalized with a 6-nitro-l-3,4-dihydroxyphenylalanine (nDOPA) anchor group to produce nDOPA-terminated PEG (nDOPA-PEG) for surface modification of MNPs using a process previously developed by Saville *et al.*,^38^ and briefly described here. The nDOPA was synthesized by nitrosating l-DOPA and precipitating the product, while mPEG-5000 was first oxidized to PEG-COOH, converted to NHS-PEG, and reacted with nDOPA in dimethylformamide. The resulting nDOPA-PEG was purified through multiple precipitation and extraction steps, then stored under inert conditions at −20 °C. To prepare MNPs for aqueous dispersibility, residual organics were removed through magnetic washes using ethanol and *n*-hexane. After ligand exchange in toluene, excess unbound ligand and organic impurities were removed using anti-solvent enhanced magnetic separation with diethyl ether and tetrahydrofuran (THF). The final PEGylated MNPs were transferred to water, filtered with a 0.22 µm PES membrane, and purified using magnetic columns to ensure removal of free ligand and aggregates.

### Induction of traumatic brain injury

A closed-head traumatic brain injury (TBI) model was used to deliver controlled mechanical impacts to mice using an electromagnetic impactor system (myNeuroLab, USA) (Fig. 1A Left panel). All procedures were approved by the University of Florida's Institutional Animal Care and Use Committee (IACUC). Male C57BL/6 mice were anesthetized with 3.0% isoflurane and placed in a prone position on a pre-warmed heating pad. Prior to impact, each animal received a subcutaneous injection of buprenorphine (0.05 mg kg^−1^) for analgesia, with subsequent doses administered every 6–8 hours for 24 hours post-procedure. The scalp was shaved using an electric trimmer, and the head was stabilized in a stereotactic frame using blunted ear bars. The impactor, equipped with a 3 mm diameter flat metal tip, was aligned over the sagittal suture between lambda and bregma, with all spatial adjustments performed manually. The Z-zero point was established by lowering the tip until it gently contacted the scalp surface, after which it was further lowered to a depth of 2.6 mm. An impact was delivered at a velocity of 6.0 m s^−1^ with a dwell time of 300 milliseconds. Time to spontaneous respiration was recorded, and all animals resumed breathing within approximately 30 seconds. Impact parameters were adopted from literature and optimized for mild-to-moderate TBI to preserve skull integrity, which mimics real-world head trauma without an invasive craniotomy.^39,40^ Mice were then transferred to a recovery cage atop a heating pad and monitored until full recovery of ambulation. All animals survived the procedure without skull fracture or external bleeding.

**Fig. 1:**
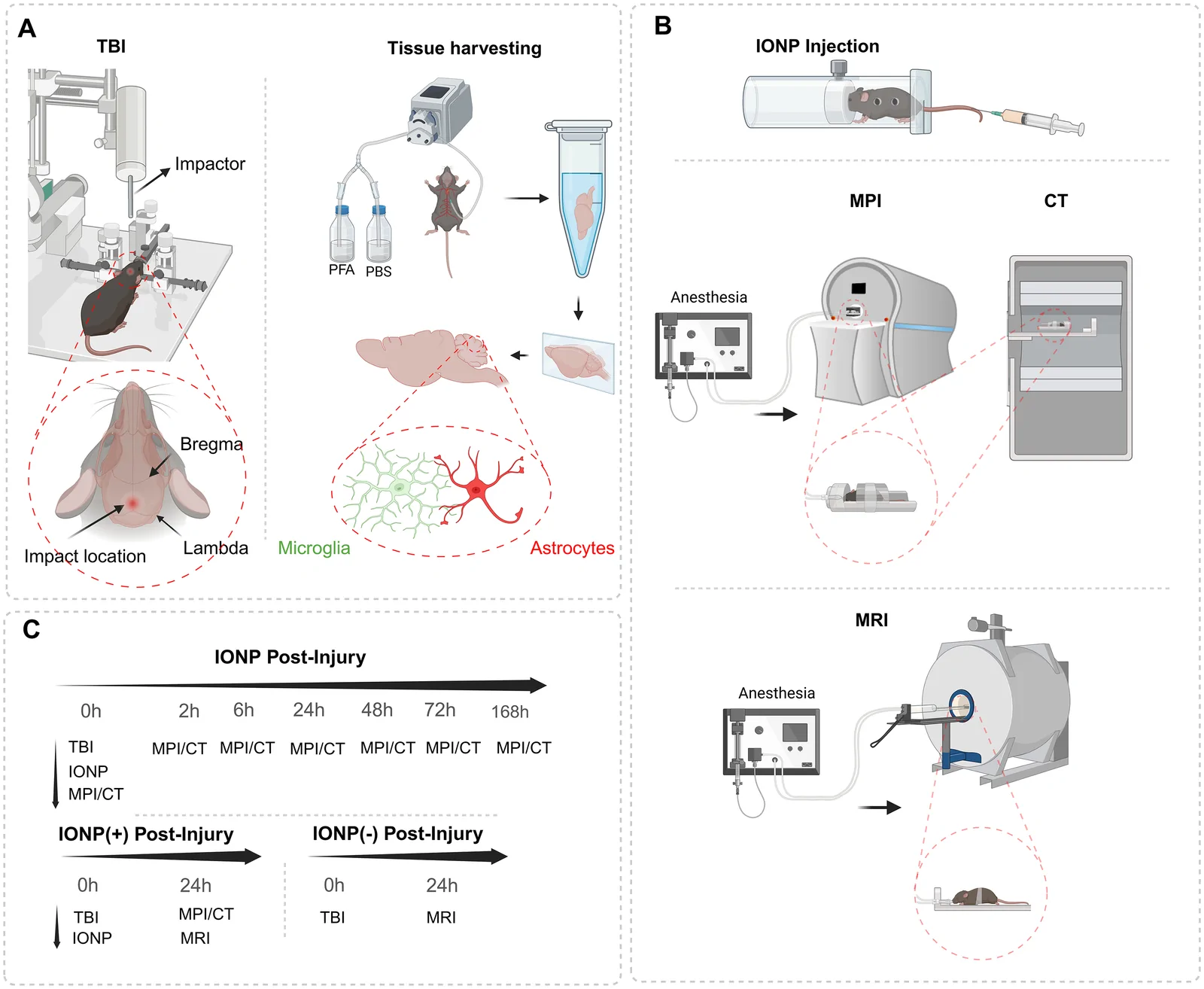
Overview of the methodology used in this study. (A) Summary of key experimental techniques: Traumatic Brain Injury (TBI) was induced using an electromagnetic impactor; transcardial perfusion was followed by brain harvesting, cryopreservation, and cryosectioning for immunohistochemistry (IHC). (B) Schematic overview of intravenous IONP administration *via* tail-vein injection and the imaging platforms used: MPI-CT (middle) and MRI (right). (C) Timeline illustrating the experimental imaging groups. Top: Closed-head TBI followed by IONP or sham control over the span of seven days. Bottom: TBI/IONP+ (TBI followed by IONP), TBI/IONP- (TBI with no IONP), Control/IOPN+ (control mice with no injury followed by IONP), and Control/IONP- (control mice with no injury and no IONP) over the span of 24 hours.

### Animal grouping for experiments

All animal procedures were conducted at the University of Florida under an approved Institutional Animal Care and Use Committee (IACUC) protocol (#202300000294). Rodent husbandry and care adhered to the University of Florida's Standard of Care for Rodents policy throughout the study. The University of Florida is accredited by AAALAC International (AAALAC #00023), holds an Animal Welfare Assurance from OLAW (D16-00244), and maintains USDA registration (58-R-0003).

Male C57BL/6 mice were used for all experiments. IONPs were administered after injury, mimicking potential clinical use. MPI/CT timelapse data and MPI/CT with MRI single time point data were generated from this study. There were two groups (*n* = 3 each) where one group received an injury, which was followed by IONPs injection *via* the tail vein approximately 15 minutes after injury, and another group served as a control where IONPs were administered, but no injury occurred. In the follow-up study comparing MPI and MRI, there were four groups (*n* = 3 each), where one group received injury followed by IONPs administered 15 minutes after TBI (TBI/IONP+), one group received IONPs with no injury (Control/IONP+), one group received injury but not IONPs (TBI/IONP−), and a final group was not given injury nor administered IONPs (Control/IONP−).

### Administration of MPI tracer

MPI tracers were administered intravenously through tail vein injections 15 minutes after TBI. Mice were allowed to recover from anesthesia, then loaded into a restraint device. The mouse's tail was isolated and placed in lukewarm water for 60 seconds to dilate the vein. The tail was removed from water, sterilized using 70% ethanol, and 100 µL of 1 mg mL_Fe_^−1^ was injected *via* the lateral tail vein using a 29G-gauge needle, resulting in a tracer dose of 100 µg_Fe_. In the event of a missed insertion, the needle was removed, then reinserted closer to the base of the tail; no animal was injected more than three times. Animal ears were punched for identification (Fig. 1B).

### Imaging procedure for magnetic particle imaging/CT

Animals were placed into an isoflurane induction box for ∼5 minutes with 4% isoflurane flowing at 1000 mL min^−1^ until under deep anesthesia, confirmed *via* toe pinch. Then, the animal was placed pronated into an in-house-built bed designed to fit both the MPI imager and the CT instrument, as shown in SI Fig. S1. Mice were anesthetized using 2% isoflurane flowing at 200 mL min^−1^ during the imaging procedure. The animal was restrained using surgical tape across the torso. The imaging procedure began with an overhead optical photo, followed by an MPI acquisition over 25 minutes. The animal was then moved to the nearby CT and imaged while still under anesthesia. After CT imaging was completed, the animal was placed on a heating pad and allowed to recover (Fig. 1B).

### MPI imaging conditions and analysis protocol

Magnetic particle imaging scans were acquired using a MOMENTUM field free line (FFL) imager (Magnetic Insight, Inc.) at the University of Florida as a series of twenty-one 2-dimensional projections using standard (Std) settings in isotropic mode. These settings had a selection field gradient of 5.7 T m^−1^, an excitation frequency of 45 kHz, and *X* and *Z* channel excitation strengths of 15.5 and 20.5 mT, respectively. The software at the time of acquisition was version 2022.1. ‘Sharpened’ image projections were collected as DICOMs from the instrument and then loaded into 3Dslicer (slicer.org^41^) for analysis.

MPI image slices and CT volumes for each time point for each mouse were loaded into 3DSlicer as volumes. The MPI projection and CT volumes were registered to one another using a previously constructed registration matrix, using four fiducial markers to create a registration matrix applied to all images. Segmentations were made in the MPI volume surrounding the liver, the tail region, and the head. This segmentation process first identified the maximum voxel intensity for each region of interest (RoI), and a minimum threshold surrounding this maximum was set at half the value of the maximum. This method was not feasible in the head region as not all animals had signals discernable from surrounding noise. Instead, a 3 mm diameter spherical segmentation (nominally 907 total voxels) was placed at the site of impact to establish a uniform RoI volume and enable comparative analysis. Total signal was calculated as the total volume of the RoI multiplied by the average signal within the RoI. This MPI total signal was used to compare animals and time points.

### Magnetic resonance imaging

Mice were scanned on a Bruker Paravision 6.0.1 controlled 11.1 Tesla MR spectrometer while sedated under a continuous paranasal flow of 1.2–1.5% isoflurane gas (delivered at 0.5 L min^−1^ mixed with medical grade air containing 70% nitrogen and 30% oxygen, Airgas, Inc.). Respiratory rates were monitored continuously, and body surface temperature was maintained using a warm water recirculation system (SA Instruments, Inc., New York). We acquired one *T*_2_ weighted anatomical and one 
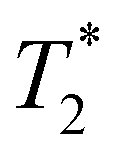
 multigradient echo sequence (MGE) per mouse. The *T*_2_-weighted Rapid Acquisition with Relaxation Enhancement (RARE) sequence was acquired with the following parameters: effective echo time = 41.4 ms, repetition time (TR) = 4 seconds, RARE factor = 16, number of averages = 12, field of view (FOV) of 15 mm × 15 mm and 0.9 mm thick slice, and a data matrix of 256 × 256 and 14 interleaved ascending coronal (axial) slices covering the entire brain from the rostral-most extent of the anterior frontal cortical surface, caudally towards the upper brainstem and cerebellum (acquisition time 9 minutes, 36 seconds). MGE images had a TR = 45 ms and 9 TE's from 2.5 ms to 26.5 ms collected in 3 ms increments, data matrix of 192 × 100 × 192 for a spatial resolution of 0.1 mm^3^. 
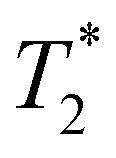
 parametric maps were generated in ImageJ using the Simplex method in the MRI processing tool, which applied a voxel-wise fitting for 
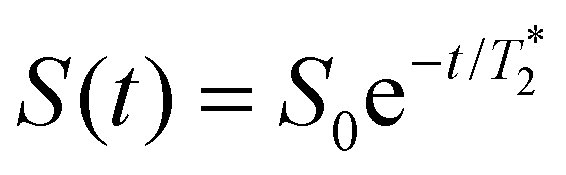
. *T*_2_ RARE images were used to identify the location of the TBI lesion. ITKSNAP was then used to manually segment lesion areas on the 
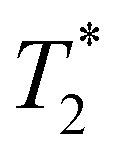
 maps and extract 
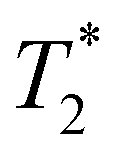
 values from various locations in and surrounding the lesion for *post hoc* analysis.

### Perfusion and brain harvesting

Animals were anesthetized using 3% isoflurane before transcardial perfusion. The heart was exposed by creating an incision in the abdominal and thoracic cavities using surgical scissors (Fig. 1A right panel). A 24-gauge blunt-end needle was inserted into the left ventricle *via* the apex of the heart and connected to a perfusion line from a peristaltic pump. Phosphate-buffered saline (PBS) was perfused at a rate of 4 mL min^−1^ for 4 minutes, followed by 4 minutes of fixative solution when applicable. After perfusion, the heads were decapitated, and the brains were carefully extracted and post-fixed in 4% paraformaldehyde (PFA) at 4 °C for 24 hours. Subsequently, the brains were rinsed in PBS and transferred to PBS containing 0.05% sodium azide for storage.

### Cryosection and immunofluorescent staining

Immunofluorescent staining was performed to assess the structural and cellular responses of the brain and to validate the severity and location of the impact (Fig. 1A). The fixed brains were then cryoprotected in 10% sucrose in PBS for 24 hours, followed by 30% sucrose in PBS for an additional 24 hours at 4 °C. Then the samples were transferred to an isopentane chamber cooled on dry ice (−80 °C) for 2 minutes. They were placed into a cryostat chamber, embedded in Optimal Cutting Temperature (OCT) compound, and sectioned at 20 μm thickness. Tissue sections were incubated in a blocking solution containing 3% goat serum and 0.3% Triton X-100 in PBS for 2 hours at room temperature. After blocking, samples were washed for 10 minutes with PBS containing 0.3% Triton X-100 (three quick rinses followed by two 2-to-3-minute washes). Primary antibodies were applied and incubated overnight at 4 °C, including anti-GFAP (AB5541, Sigma Aldrich) for astrocyte labeling and anti-Iba1 (019-19741, Wako Chemicals) for microglial detection. After a 10 minute wash, secondary antibodies, goat anti-chicken IgY conjugated with Alexa Fluor 633 (A21103, Life Technologies) and goat anti-rabbit IgG conjugated with Alexa Fluor 488 (111-545-144, Jackson ImmunoResearch), were applied for 2 hours at room temperature. Following this, Hoechst nuclear stain was added for 15 minutes, and the sections were washed again for 10 minutes before being mounted with VECTASHIELD mounting medium. Images were acquired using a Keyence BZ-X810 fluorescence microscope (Keyence Corporation, Japan).

### Statistical analyses

An *a priori* power analysis was performed using G*Power version 3.1.9.7 to estimate the effect size required to detect a difference between independent groups. Analysis was conducted with an alpha level of 0.05, statistical power of 0.95, and sample size of *n* = 3 per group. Under these assumptions, the minimum detectable effect size was Cohen's *d* = 4.02. The following statistical tests were performed in GraphPad Prism. For Fig. 4B, one-phase decay models were fit to the datasets, represented by solid lines, and the respective 95% confidence intervals are represented with dashed lines. For the panels in Fig. 5D, ordinary one-way ANOVA tests followed by Tukey's multiple comparisons test were performed to compare groups, specifically the Control/IONP+ and TBI/IONP+ groups to one another and the TBI/IONP− and TBI/IONP+ groups to one another. Significance was defined as a *p*-value less than 0.05. An unpaired *t*-test was also performed for Fig. 5E using Graphpad Prism.

## Results

### Histological validation of the controlled closed-head traumatic brain injury model

To validate the injury location and associated tissue response following closed-head TBI, IHC was performed on brain sections harvested 7 days post-injury. Fig. 2 shows representative immunofluorescence images targeting three key markers: Iba1 (green) for microglial activation, GFAP (red) for astrocyte reactivity, and Hoechst (blue) for nuclear staining.^42^ Compared to the sham group, the TBI group exhibited significant microglial activation at the impact site, indicated by dense Iba1-positive cells localized within the cerebellar region. Activated microglia displayed hypertrophic, bushy morphologies characteristic of reactive microglial responses following CNS injury.^42^ While the highest density of microglial activation was concentrated near the impact site, surrounding regions also demonstrated mild activation (Fig. 2, Iba1 TBI). GFAP staining revealed reactive astrocytosis in the cerebellar region, with post-injury astrocytes demonstrating enhanced signal intensity and thickened cellular processes. The observed injury pattern remained anatomically localized despite the 3 mm impactor size and involved disruption of the molecular, Purkinje, and granule cell layers (SI Fig. S2). Prussian Blue staining was also performed to assess iron deposition; however, no detectable staining was observed in the injured brain tissue under the conditions evaluated.

**Fig. 2:**
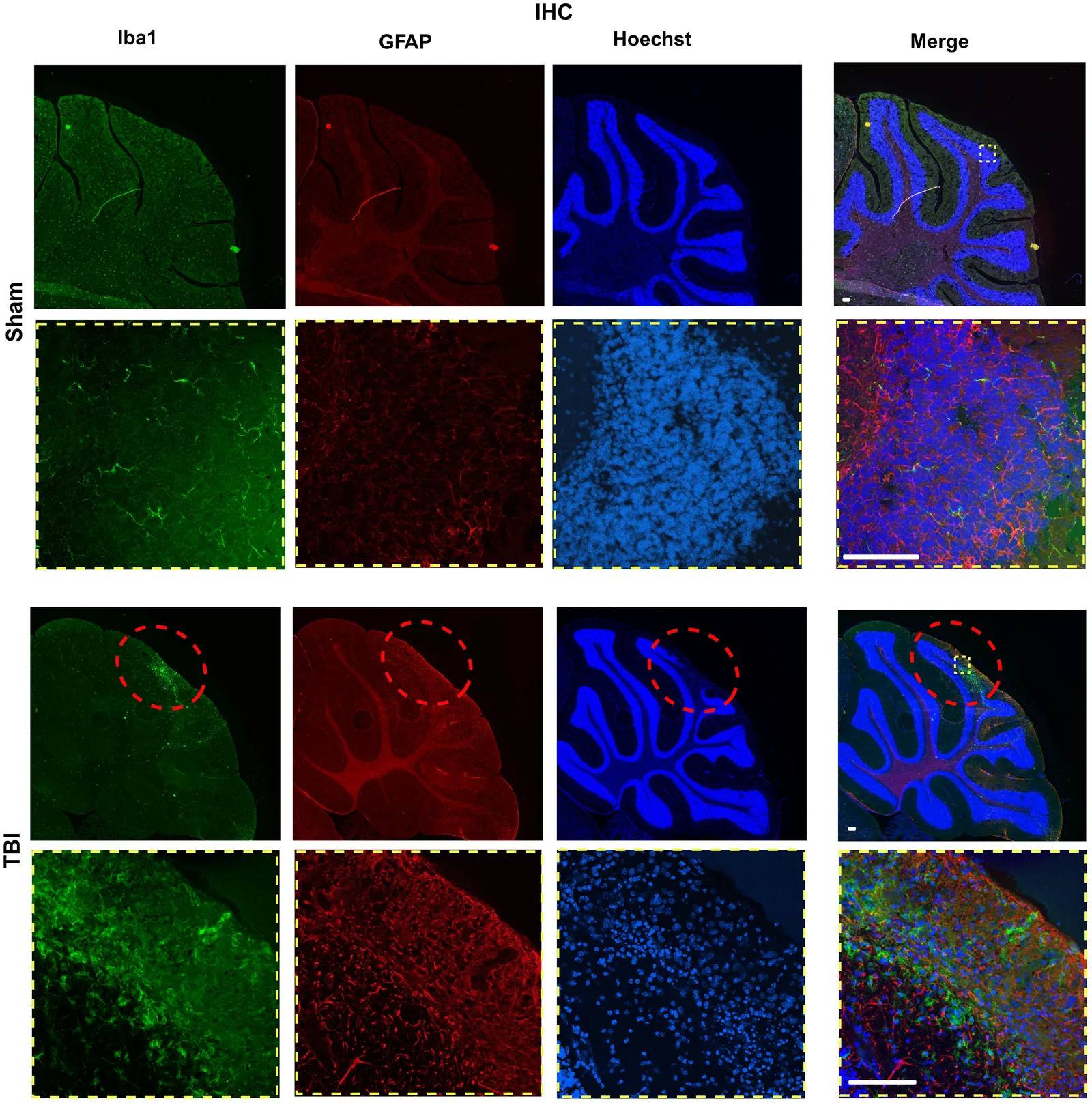
Immunohistochemical validation of injury localization following closed-head TBI. IHC staining was performed to assess structural and immune responses, including astrocytes (GFAP, red), nuclei (Hoechst, blue), and microglia (Iba1, green), to validate the injury location and severity. Red-dash circles indicate the injury site, localized in the cerebellar region. Images are representative of the TBI group at 7 days post-injury. Scale bar: 100 µm.

### IONP characterization

Magnetic nanoparticles were synthesized *via in situ* oxidation during thermal decomposition and coated with PEG using a catecholamine anchoring group.^33,43,44^ The resulting IONPs had iron oxide cores with diameter of 21.8 ± 2.9 nm through TEM (SI Fig. S3), hydrodynamic diameters with a log–normal fit of the number weighted distribution to be 44.5 nm with ln *σ* = 0.10 according to dynamic light scattering (SI Fig. S4), where the number weighted distribution has a polydispersity index (PDI) of 0.01. Additionally, the IONPS had estimated magnetic diameters of 20.8 ± 0.2 nm from magnetic characterization, an MPI signal of 169 ± 2 mg_Fe_^−1^, and full-width-at-half-maximum (FWHM) resolution of 8.7 ± 0.2 mT according to an average of five measurements in the RELAX module of the MOMENTUM™ Imager (Magnetic Insight, Inc.) (SI Fig. S6). As a reference, characterization of the commercial tracer VivoTrax conducted in the same instrument and with the same conditions, resulted in a point spread function with an MPI signal of 38.5 mg_Fe_^−1^ and a full-width-at-half-maximum (FWHM) resolution of 10.36 mT.^45^

### Visualization of TBI using magnetic particle imaging

Fig. 3 summarizes key observations for this study. In all images, a skeleton segmentation extracted from CT scans is shown in aqua blue as a maximum intensity projection, while the MPI signal is a slice view centered on the maximum intensity in the region of interest. The TBI groups had shown a strong MPI signal in the head region in the scan immediately after impact and tracer administration, while control animals did not. Representative animals are depicted in Fig. 3A. In this panel, contour lines were constructed to visualize the distribution of signal. The color scale shown in Fig. 3A, from 0.04–0.09 a.u., was used for all images in the figure, regardless of condition or time point, to allow ease of comparison. Additional images for representative mice using other color scales are shown in SI Fig. S4. This signal in the injury site can be examined further in Fig. 3B, where each panel depicts different views of the MPI/CT 3D volume overlay. The 3D projection (top right) in Fig. 3B illustrates two strategies for signal quantification. An orange mask corresponds to the volume defined by signal above 50% of the maximum value in the injury site, while the pink mask corresponds to a 3 mm diameter sphere (nominally 907 total voxels) positioned with its center in the expected site of injury. Both approaches are possible when the MPI signal at the site of injury is easily differentiated from the MPI signal from major blood vessels and organs that accumulate nanoparticles, as illustrated in Fig. 3A using contour lines for the TBI case. However, defining a mask based on signal thresholds fails when the signal is not easily differentiated, as would be the case for the control mouse illustrated in Fig. 3A. In contrast, use of a consistently positioned constant volume sphere is possible in all mice and for all time points. This is further illustrated for the TBI and control mice in Fig. 3C and D, which show whole-body CT of the animal skeleton with masks defined using 50% threshold of the signal at the injury site (orange, only in Fig. 3C for the TBI mouse), 3 mm diameter sphere (pink), 50% threshold of the signal in the liver (green), and 50% threshold of the signal in the site of administration in the tail (blue), the latter of which illustrates residual signal due to IONP tissue entrapment during injection. These animals were imaged over a span of 7 days, and the signal decayed over time, as shown visually for a representative animal from each group in Fig. 3E. As time progressed, the MPI signal in the brain region reduced to below 0.04 a.u.

**Fig. 3:**
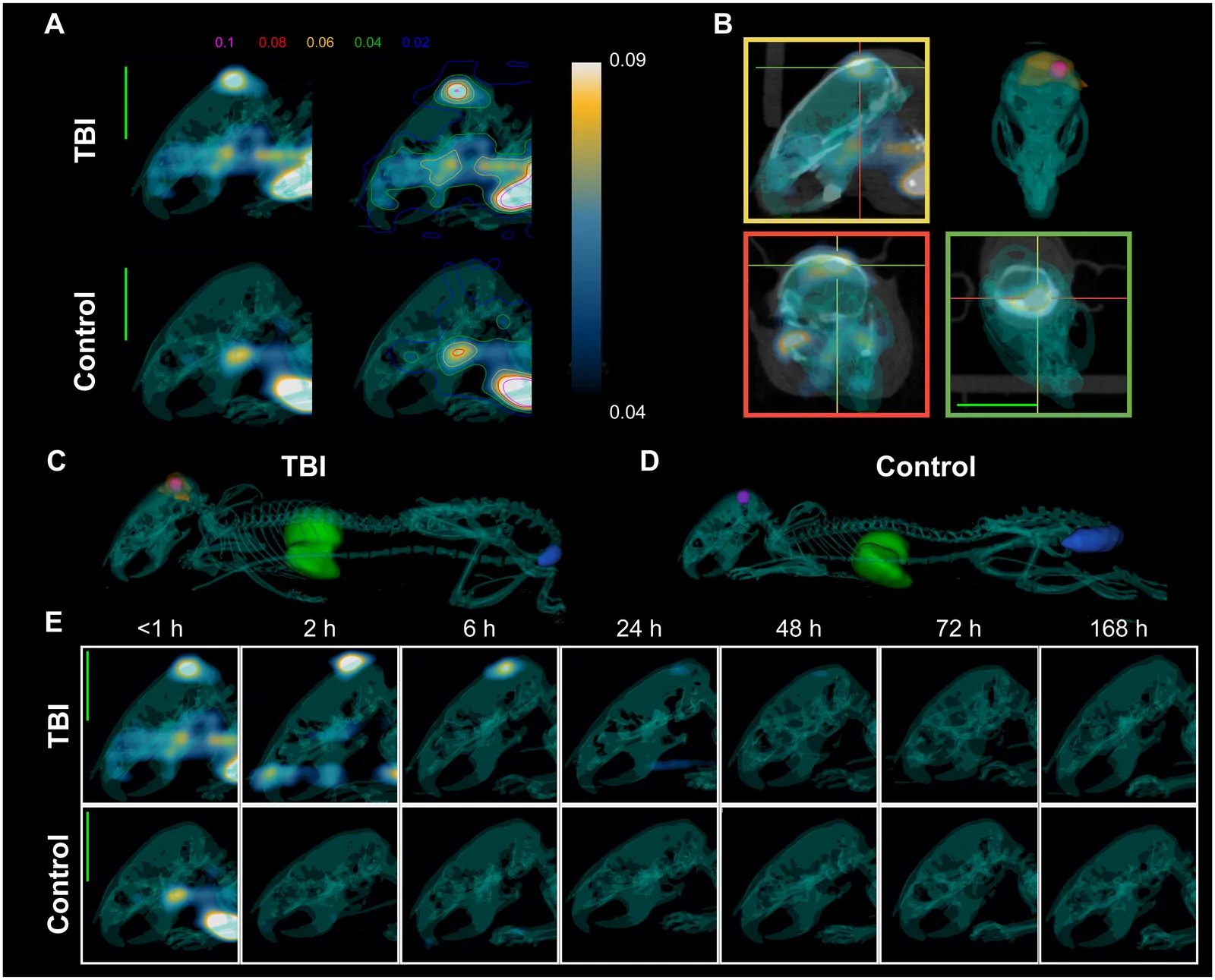
Magnetic particle imaging enables tomographic visualization of traumatic brain injury. (A) Sagittal combined MPI and CT head images of TBI animals that received IONP (top) and control mice that received IONP but not injury (bottom). The images on the left show the MPI and CT scans, while the images on the right show contour lines of MPI signal intensity according to the color code key above the images. The contour lines clearly demonstrate signal separation between the site of TBI and major blood vessels in mice that received injury, while there is no signal separation for the top of the skull in the control mice that did not receive injury. The color bar was used for all images in the figure. (B) Axial (red border), 3D (no border), sagittal (yellow border), and coronal (green border) views of an MPI scan of a representative mouse after injury. Vertical and horizontal lines within each view correspond to the planes through which the corresponding alternate views were taken. In the 3D panel, the orange mask corresponds to 50% of the maximum signal within the region, while the pink mask corresponds to a 3 mm sphere (nominally 907 total voxels) centered at the injury site. These masks are further illustrated in (C) for a mouse that received IONP prior to TBI and (D) for a mouse that received IONP but no injury. In the former, masks can be defined based on 50% of the maximum signal at the injury site and using the painted sphere, but in the latter, a mask can only be defined using the painted sphere (pink). In both, masks are also shown corresponding to 50% of the maximum signal in the liver (green) and in the site of tail vein injection (blue). (E) Timelapse of head sagittal images for one representative animal from each group showing signal decay over time.

### Quantification of magnetic particle imaging signal in TBI

Next, we quantified the MPI signal in the brain as a function of time for control and injured mice (*n* = 3, each) that received IONP after injury. Because the MPI signal at the top of the head (the injury site in the TBI animal groups) could not be easily differentiated for all groups and at all time points, we used the total MPI signal contained within the 3 mm diameter spherical masks introduced in Fig. 3, placed in the calvarial region of the brain. Fig. 4A depicts a representative animal where particles were administered after injury. In the images shown in Fig. 4A, a strong MPI signal can be seen in the calvarial region of the brain, near the site of impact. However, this is not the case for all time points in the injury group and is not the case for all animals in the control group, as illustrated in SI Fig. S4. The total MPI signals inside the spherical mask for TBI and control animals over the 7 day study period are shown in Fig. 4B. A one-phase decay model was fitted to the data, resulting in a clearance half-life of 23.9 hours for the group that received IONPs post-TBI, and 2.1 hours for the control group.

**Fig. 4:**
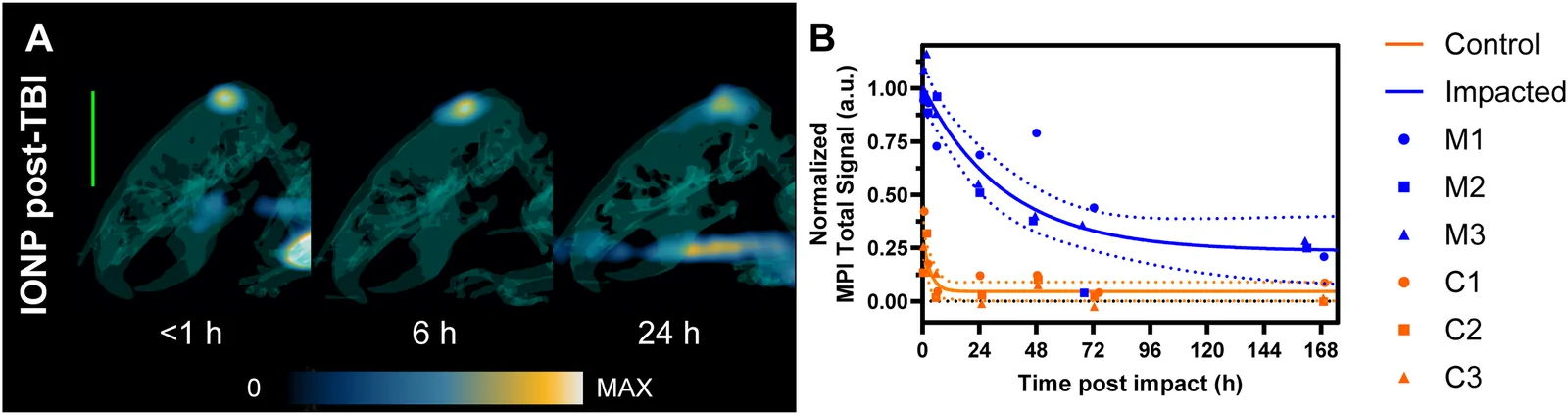
Magnetic particle imaging enables non-invasive quantitative evaluation of nanoparticle accumulation and clearance from sites of traumatic brain injury. (A) MPI/CT combined images for a representative animal that received IONP approximately 15 minutes after injury. Included time points are at less than an hour after IONPs administration, 6 hours after IONPs administration and 24 hours after IONPs administration. The signal in each image has been scaled between the maximum and minimum signal for the region in the corresponding animal and time point. (B) Total MPI signal inside the 3 mm spherical mask normalized to the mean of the initial values of the three mice in the impacted group, showing slower signal decay for the impacted group relative to the control group in both studies. A one-phase decay model fitted to each group, represented by solid lines, and 95% confidence interval, represented by dashed lines, suggests a clearance half-life of 23.9 hours for the group that received IONPs post-TBI, and 2.1 hours for the control group. Time is measured from the impact for TBI animals and from the IONP injection for control animals; TBI animals received IONPs approximately 15 min after impact.

### Magnetic particle imaging and magnetic resonance imaging

Next, we sought to compare the potential of MPI and MRI to differentiate injured and control mice at the 24 hours time point. The study included four groups of *n* = 3 mice (Fig. 1C, bottom): mice that received TBI followed by IONP administration (TBI/IONP+), mice that received IONP but no injury (Control/IONP+), mice that received TBI but did not receive IONP (Control/IONP−), and mice that did not receive injury or IONP (Control/IONP−). All mice were imaged using *T*_2_-weighted Rapid Acquisition with Refocused Echoes MRI (*T*_2_ RARE MRI) and 
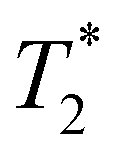
 multigradient echo sequence (MGE) at 24 h, while only mice that received IONP were imaged by MPI/CT at 24 h. Representative images are organized in columns in Fig. 5A–C for mice that received IONP with no injury (Control/IONP+, open symbols) in the left column and for mice that received injury and IONP (TBI/IONP+, with closed symbols) in the right column. Structural MRI (*T*_2_ RARE) images are shown in Fig. 5A, 
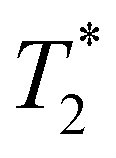
 weighted MRI images are shown in Fig. 5B, and MPI images are shown in Fig. 5C. The panels in Fig. 5C are co-registered MPI and CT slices located at the maximum intensity for MPI in the region of the injury. In Fig. 5A, a signal void can be seen in the *T*_2_-weighted RARE images of impacted mice, consistent with an injury to the brain. This imaging sequence was acquired for structural imaging only, and while it can be optimized for quantification, the study was designed to use *T*_2_ RARE MRI for identification of the lesion and to use 
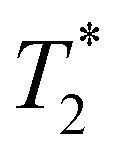
 MRI for quantification. In Fig. 5B the 
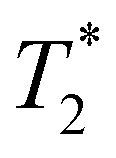
 MRI, thresholded from the minimum to the maximum voxel value within the RoI, does not show any discernable difference between the control and impacted mice. In Fig. 5C, a clear difference in the MPI signal, thresholded from the minimum to the maximum voxel value within the RoI, can be seen when comparing images of the impacted and control mice. Quantification of 
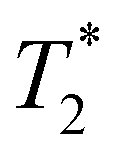
 MRI was performed in the cortex, ipsilateral, contralateral, and muscle regions of the animal using ITKSNAP and is depicted in Fig. 5D. No significant differences were found when comparing the animals with injury and no particles (TBI/IONP−) to the animals with injury and particles administered (TBI/IONP+) and when comparing animals without injury and particles administered (Control/IONP+) to animals with injury and particles administered (TBI/IONP+). In contrast, MPI signals, depicted in Fig. 5E with peak signal-to-noise ratio and total signal, show significant differences (*p* = 0.0008 and *p* = 0.0006, respectively) when comparing the control/IONP+ and TBI/IONP+ groups.

**Fig. 5:**
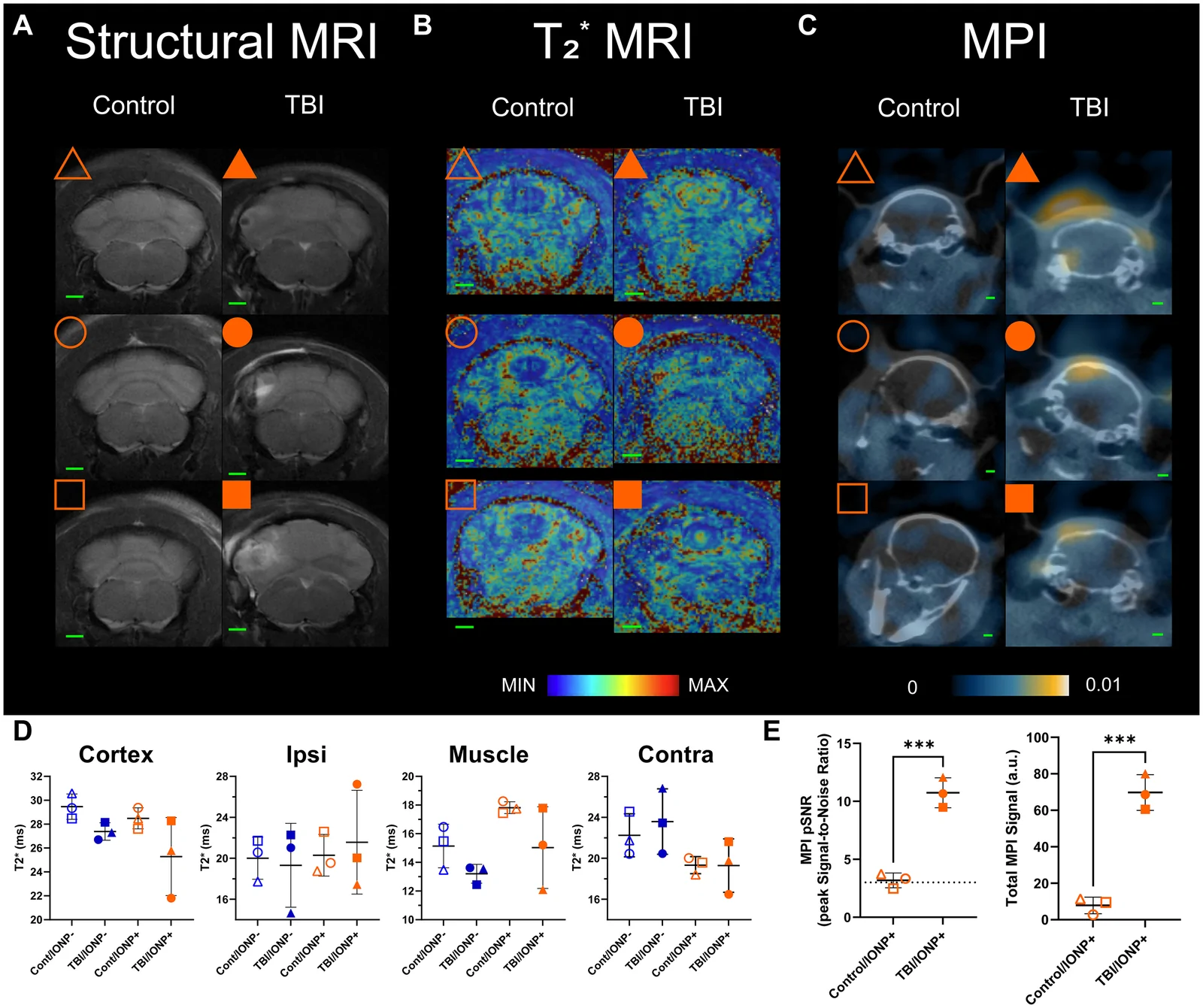
Magnetic particle imaging outperforms magnetic resonance imaging in identifying traumatic brain injury. Mice received injury or sham followed by IONP administration at approximately 15 minutes and were imaged using MPI/CT and MRI the next day. (A) *T*_2_ RARE MRI and (B) 
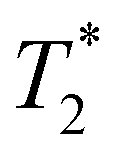
 parametric map derived from multi-gradient echo MRI and (C) MPI/CT slices located at maximum intensity for MPI in the region of the injury for control (open symbols) and injured (closed symbols) mice 24 hours after IONP administration, where the overlaid symbols correspond to mouse number and are consistent across all plots. Structural MRI in the TBI mice shows signal enhancement in the cerebellar simple lobule area, away from the site of injury. 
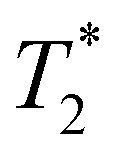
 maps do not show evidence of changes caused by injury or by IONP accumulation. MPI clearly shows signal accumulation at the site of injury in all mice of the TBI group. (D) 
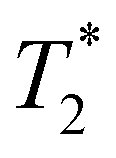
 measures in specific sections of the brain, with closed symbols corresponding to TBI groups, open symbols corresponding to control groups, blue markers corresponding to animals without IONP administered, and orange markers corresponding to animals with IONP administered. A one-way ANOVA test was performed, and the statistical comparisons are multiple comparisons of groups. No statistically significant differences were observed between groups for any region examined, suggesting 
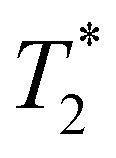
 MRI is unable to distinguish control and injured mice, with and without IONP administration. (E) Comparison of MPI pSNR and total MPI signal within the mask defined using the 3 mm sphere for groups administered IONPs, with tie bars representing an unpaired *t*-test (*** corresponds to *p* < 0.001). The dotted black line represents pSNR = 3.

## Discussion

A calibrated electromagnetic impactor system was used to deliver controlled mechanical impacts to the sagittal suture between lambda and bregma to induce closed-head TBI. This model provides controlled specifications of impact velocity, compression depth, dwell time, and impactor tip geometry. Unlike the approach of Orendorff *et al.*,^32^ which used a weight-drop device, the electromagnetic impactor controls each parameter independently, producing consistent and reproducible injuries across animals. This better reflects how real concussive injury occurs and avoids the inflammation and vascular changes that craniotomy-based models, such as controlled cortical impact and fluid percussion injury can introduce.^46^ None of the TBI animals developed skull fractures or external bleeding, confirming that the impact parameters produced a mild-to-moderate injury as intended.^39^ To validate the injury, brains were harvested at 7 days post-injury. This time point was chosen because microglial and astrocytic activation are most visible during this subacute phase following closed-head trauma.^47^ IHC images showed a focal neuroinflammatory response at the impact site. Microglia displayed hypertrophic morphology, and astrocytes showed increased GFAP expression, both of which are well-established markers of the glial response to TBI brain injury in rodent models.^42,48^ These changes reflect active secondary injury rather than recovery. Collectively, the controlled impact conditions and the histological findings confirm that the model produces a focal, reproducible, mild-to-moderate injury detected by MPI.^47,48^ In these studies, nanoparticles were administered intravenously *via* the tail vein to mice after TBI. MPI and CT scans were collected using the MOMENTUM™ imager and an IVIS Spectrum/CT instrument located in adjacent rooms, with mice placed in a custom-designed bed compatible with both instruments. This dual imaging modality setup allowed acquisition of images using both modalities without disturbing the mouse, thereby facilitating multi-modal image registration (Fig. 1B). Registration between MPI, CT, and MRI scans was not feasible because the MRI scanner is in a separate building, and MPI/CT and MRI animal beds are not currently compatible. The co-registration of MPI and MRI would have allowed a more specific and MPI-guided analysis of 
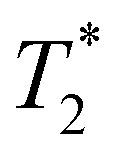
, allowing direct comparisons between modalities.

As a result of their size, systemically administered nanoparticles often remain confined within blood vessels and only extravasate at sites of vascular injury or within organs that actively remove foreign material from the bloodstream. These features make nanomaterial technologies such as MPI attractive for the precise diagnosis and treatment of brain injury resulting in changes in cerebral blood perfusion and hemorrhage.^29,49,50^ Prior studies have reported the potential MPI in imaging changes in cerebral blood perfusion during acute stroke,^29^ hypercapnia,^51,52^ intracranial hematoma,^53^ and traumatic brain injury.^32^ Ludewig *et al.*^54^ demonstrated the potential of MPI in imaging ischemic stroke by monitoring transient perfusion of a bolus of MPI tracer in a mouse model of severe and acute ischemic stroke.^29^ Mason *et al.*^51^ evaluated the potential of functional MPI using a rat model of hypercapnia, where changes in cerebral blood perfusion were induced by supplying the animal with alternating cycles of normal and CO2-enriched air. Owing to its high sensitivity and specificity, MPI measures of changes to cerebral blood volume (CBV) resulted in contrast-to-noise ratios (CNR) outperforming 9.4 T BOLD functional MRI, establishing MPI as a sensitive, positive-contrast CBV-based neuroimaging modality. This study was recently followed up by Barskdale *et al.*,^52^ who reported initial results of monitoring changes in CBV induced by hypercapnia in non-human primates using a human-capable MPI scanner.^55^

Szwargulski *et al.*^53^ demonstrated MPI detection of intracranial hematoma, with real-time monitoring of hematoma expansion and use of multi-contrast MPI to differentiate regions of liquid and coagulated blood. They used a model of severe intracranial hemorrhage induced *via* injection of collagenase directly into the striatum, and the tracer was administered 30 minutes after inducing the hematoma. Two tracers were evaluated with different circulation times. Using digital image subtraction, the authors delineated regions of hemorrhage with the longer circulating tracer. MPI and MRI measurements of the volume over which bleeding had occurred were similar in this model of extensive intracranial bleeding. Mice were euthanized 1 hour after inducing hemorrhage, precluding study of long-term accumulation and clearance of the MPI tracer from the brain. While Szwargulski *et al.*^53^ evaluated MPI in the context of intracranial hemorrhage, their intracranial collagenase injection mouse model is not representative of traumatic brain injury, where damage arises from external forces. Orendorff *et al.*,^32^ published in 2017, is the only study to date exploring the use of MPI in the context of TBI. Although these authors illustrated the potential use of MPI in the context of TBI, their pilot study included a single rat subjected to injury. Furthermore, injury was caused using a lab-built weight-drop impact device that was not validated to assess the extent of injury severity. While weight-drop impact devices are widely used to model TBI, they are prone to high variability even when using the same nominal parameters, because they do not provide precise control over the location and extent of injury.^56^ In contrast, our study used a well-validated model of TBI consisting of a stereotaxic apparatus and an electromagnetic impactor system that enables precise control of the site of injury and of impact area, force, velocity, dwell time, and compression depth, all of which result in controlled and reproducible brain injury that mimics concussive head injury with well-characterized progression of changes in brain morphology and biochemical markers.^34^ In contrast to Orendorff *et al.*,^32^ our study evaluated MPI tracer accumulation at the injury site in cohorts of mice that received tracer after injury, a more clinically relevant scenario. We observed clearly differentiated signal accumulation in the injury site of animals in the TBI groups, without the need for digital signal subtraction used in other studies of MPI in cerebral injury. Additionally, our model of controlled moderate TBI allowed us to study tracer accumulation and reduction over a period of 7 days, with observation of extended retention of signal in the region of injury that allowed high confidence in differentiating control and injury groups even 24 hours after injury. Finally, we found that MPI could reliably delineate the site of injury through tracer accumulation at 24 hours after injury, whereas 
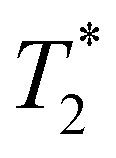
 weighted MRI could not. It is important to note that while MPI uses exogenous contrast to enhance IONPs signal from the injury site, 
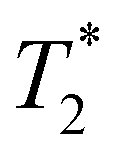
 relies on intrinsic chemical relaxation rates subject to a variety of factors within and beyond the site of injury.

While our study demonstrated the potential capabilities of MPI to detect TBI, there were several limitations. These studies did not explore the effects of extent of injury (*i.e.*, mild *vs.* moderate *vs.* severe) and the associated transient blood brain barrier opening on MPI signal and nanoparticle accumulation and clearance. Future studies are also needed to determine the mechanisms responsible for nanoparticle recruitment and retention at sites of traumatic brain injury. The role of nanoparticle physicochemical properties (*e.g.*, size and charge) and nano–bio interactions (*e.g.*, protein corona formation, use of targeting moieties, and nanoparticle uptake by circulating and resident immune cells) would further enhance knowledge of modulating nanoparticle accumulation and clearance following a traumatic brain injury. While performing *a priori* statistical power analysis to confirm our cohort size could detect reliable MPI signal, we did not power the study to directly compare MPI and MRI. Our goal was to determine whether MPI could detect differences between the injured and control mice, which we succeeded in doing. To determine the statistical significance of differences in MRI signal between cohorts, an *a posteriori* sample size analysis was performed *via* a two-tailed *t*-test with an alpha error probability of 0.05, statistical power of 0.95, and calculated effect size. This test indicated we would need a relative cohort size of 13 animals to evaluate these differences in the cortex and temporalis muscle regions, 400 animals to evaluate the difference in the ipsilateral region, and over 45 000 to evaluate the differences in the contralateral region. In contrast, for the MPI group, statistical power analysis with an alpha level of 0.05, statistical power of 0.95, and a sample size of *n* = 3 per group, suggested a minimum detectable effect size of Cohen's *d* = 4.02, which is smaller than the observed Cohen's *d* of 8.21.

## Conclusions

Traumatic brain injuries are difficult to diagnose and can lead to serious complications if left untreated. CT and MR imaging are current standards for first response to TBI, which have limitations regarding specificity and resolution for brain bleeding, especially for mild and moderate injuries. The study described here demonstrates that magnetic particle imaging is a powerful tool for detecting TBI and quantifying nanoparticle accumulation and clearance from sites of injury, non-invasively and longitudinally, with high specificity and sensitivity, when the nanoparticle tracer is administered after injury. Additionally, comparison between MPI and MRI measures of TBI and IONP accumulation demonstrates the former's superior ability to detect brain bleeds following trauma with improved specificity and resolution. The results presented here illustrate the tremendous potential of MPI as a tool to evaluate these phenomena, and to serve as a tool for evaluating and developing drug carriers for treatment of TBI. Further, considering the rapid recent development of clinical-scale MPI scanners suitable for use in the human brain,^55,57–60^ the studies described here support the tremendous potential for MPI as a clinical tool in early detection of TBI.

## Author contributions

Hayden J. Good conducted the TBI and MPI experiments, performed MPI analysis, analyzed results, and wrote the manuscript. Mohammad Tabatabaei conducted the TBI Experiments, performed IHC and fluorescence imaging, analyzed results, and wrote the manuscript. Eiko Alzamora assisted in TBI and MPI experiments. Neel Eswaran and Sk Rajab Ali performed nanoparticle surface coating experiments and conducted characterization of said nanoparticles. Ambar C. Velazquez-Albino performed nanoparticle synthesis experiments. Zachary Simon conducted MRI experiments. Marcelo Febo conducted MRI experiments, performed MRI analysis, and assisted in writing the manuscript. Lakiesha Williams conceived the experiments and assisted in interpretation of data. Carlos M. Rinaldi-Ramos conceived the experiments and assisted in interpretation of data. All authors have read and edited the manuscript.

## Conflicts of interest

CRR is an inventor on patents (US 10,634,742 B2, US 10,765,744 B2, US 11,305,351 B2, US 11,311,630 B2, US 12,072,400 B2) and patent applications (US 2022/0287969 A1, US 2023/0355811 A1), which are awarded or submitted in whole or in part to the University of Florida and are related to magnetic nanoparticles or magnetic particle imaging. The university may benefit financially from their commercialization, and the author could benefit under the university’s patent policy. All other authors declare that they have no other competing interests.

## Abbreviations

MPI: Magnetic particle imaging
MRI: Magnetic resonance imaging
TBI: Traumatic brain injury
IONPs: Iron oxide nanoparticles
CCI: Controlled cortical impact
CT: Computed tomography
nDOPA: 6-Nitro-L-3,4-dihydroxyphenylalanine
THF: Tetrahydrofuran
IACUC: Institute of animal care and use committee
FFL: Field free line
MGE: Multigradient echo
TR: Repetition time
FOV: Field of view
RoI: Region of interest
RARE: Rapid acquisition with relaxation enhancement
PBS: Phosphate-buffered saline
PFA: Paraformaldehyde
OCT: Optical cutting temperature
IHC: Immunohistochemistry
FWHM: Full-width at half-maximum
PEG: Poly(ethylene glycol)
pSNR: Peak signal-to-noise ratio
mSNR: Mean signal-to-noise ratio

## Supplementary Material

NR-018-D6NR02497A-s001

NR-018-D6NR02497A-s002

## Data Availability

Data for this article are available at Dryad at https://doi.org/10.5061/dryad.6t1g1jxdz. Supplementary information (SI) is available. See DOI: https://doi.org/10.1039/d6nr02497a.
