# Supplementary material for: Sensitive detection of in vivo traumatic brain injury foci using magnetic particle imaging
Source: Nanoscale. 2026 Sep 24. Online ahead of print. doi: 10.1039/d6nr02497a (PMC13721992; doi:10.1039/d6nr02497a)
Supplement: Supplementary file 1 [file NR-OLF-D6NR02497A-s001.pdf]

Supplementary Information (SI) for Nanoscale.
This journal is © The Royal Society of Chemistry 2026

**Supplementary Material for "Sensitive Detection of In Vivo Traumatic Brain Injury using Magnetic Particle Imaging"**

**Authors:** Hayden J. Good[1,*], Mohammad Tabatabaei[2,*], Eiko Alzamora[2], Neel Eswaran[1], Sk Rajab Ali[1,2], Ambar C. Velazquez-Albino[1], Zachary Simon[3], Marcelo Febo[3], Lakiesha N. Williams[2,-], and Carlos M. Rinaldi-Ramos[1,2,†]

1. Chemical Engineering, University of Florida, 1075 Center Dr, Gainesville, FL, 32611
2. J. Crayton Pruitt Department of Biomedical Engineering, University of Florida, 1275 Center Dr, Gainesville, FL, 32611
3. Department of Psychiatry, McKnight Brain Institute, University of Florida, 1149 S. Newell Dr, Gainesville, FL, 32611

* These authors contributed equally.
[†]Co-corresponding authors

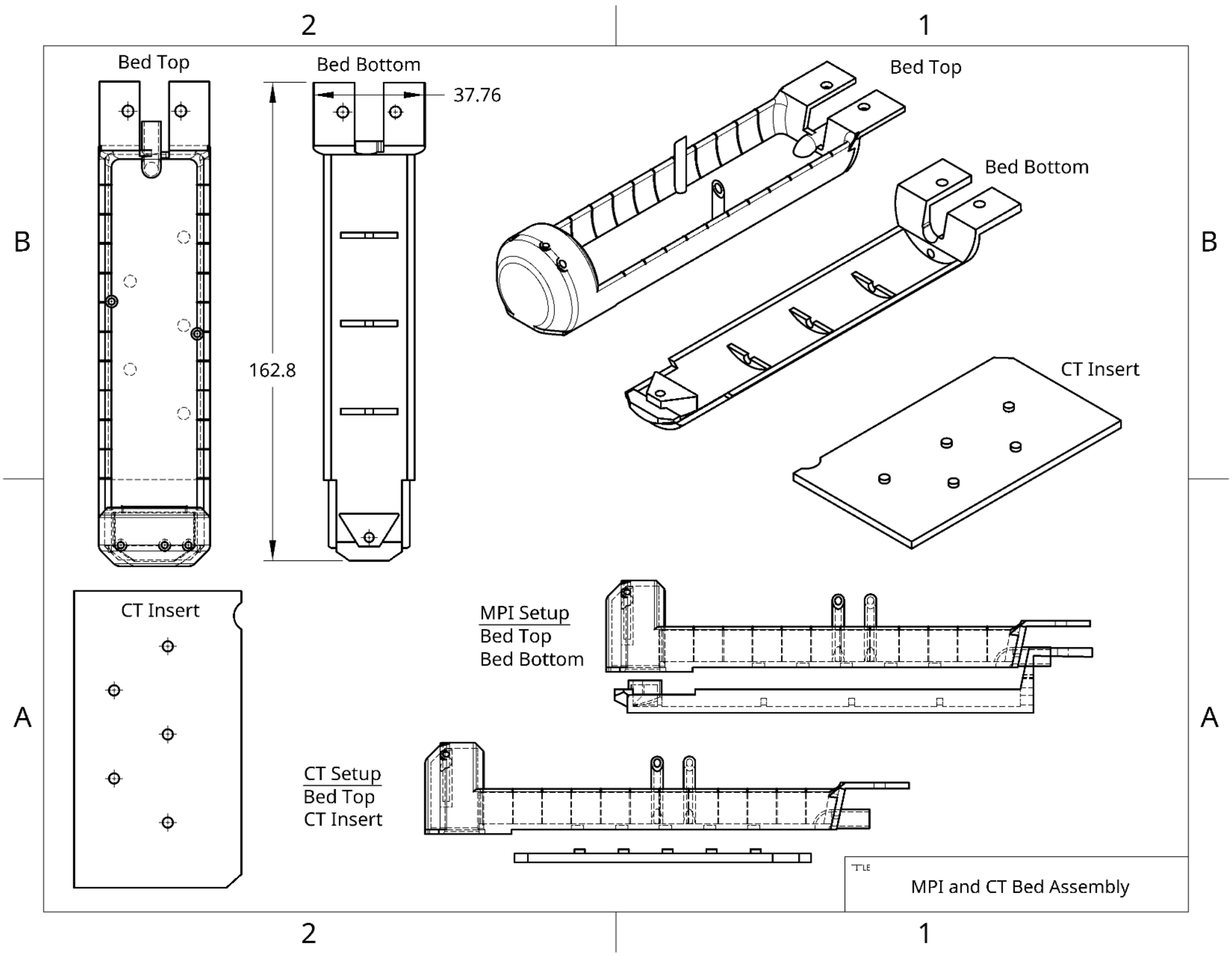


**Supplementary Figure 1: CT and MPI Bed Assembly Drawing.** A customized animal bed was utilized during the study. Three pieces were used, the Bed Top, Bed Bottom, and CT Insert. The Bed Top and Bed Bottom were used to image the animal under anesthesia in the MPI. The Bed Top, where the animal rested, was then disconnected from the Bed Bottom, and brought to the CT machine, connected to the CT Insert using the guide pegs, then imaged at the CT under anesthesia.

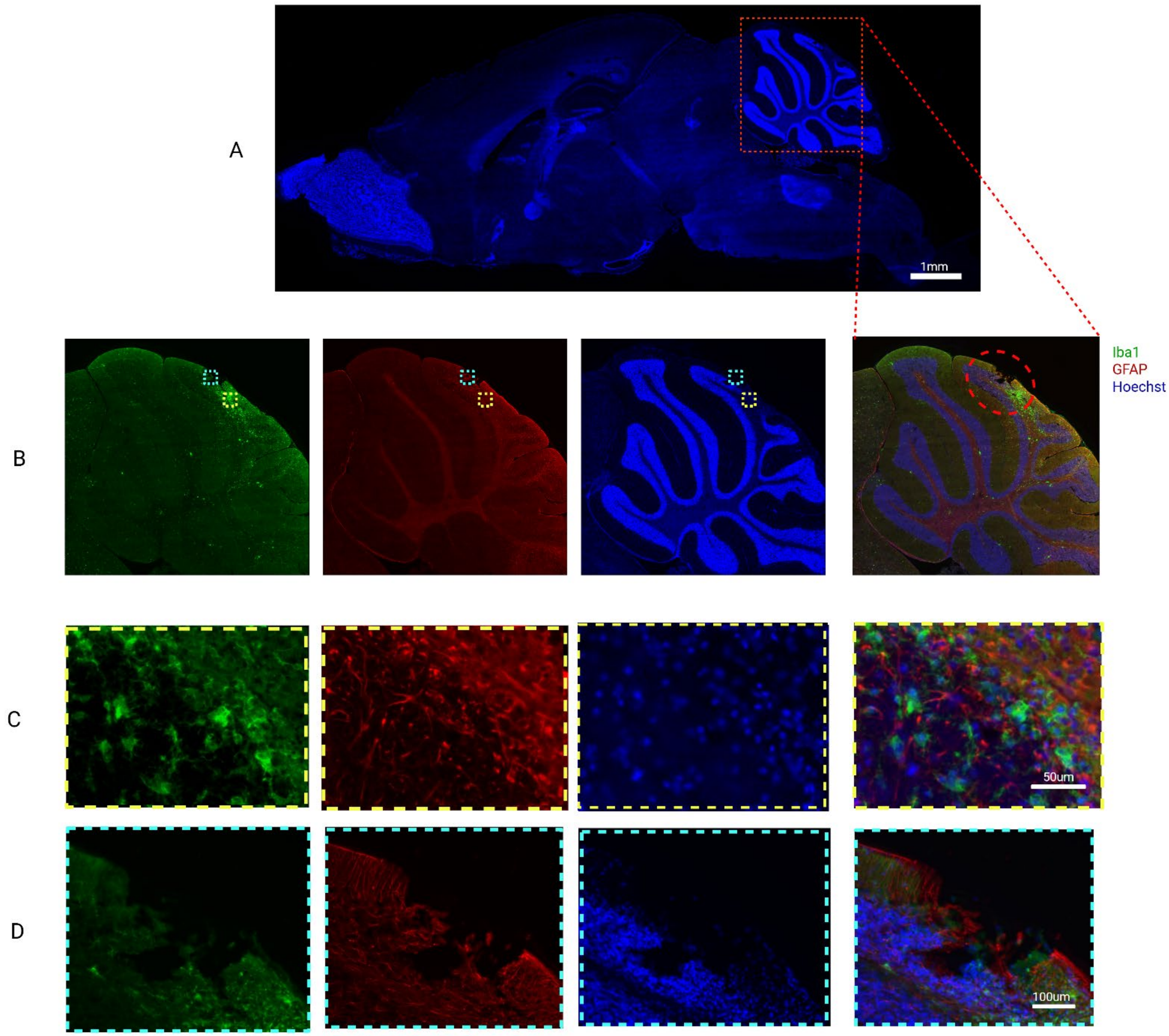


**Supplementary Figure 2**: Immunofluorescent images of TBI mice after 7 days post impact. IBA in green, GFAP in red, and Hoechst in blue. A) Sagittal section of impacted mice after 7 days. B) magnified image of the impacted region, which occurred in the cerebellum. Dashed circle shows the exact impacted region C) magnified the region clearly showed IBA1 activation in which cell morphology and Iba1 signal increased compared to nearby proximity. These activated microglia exhibit hypertrophic, bushy morphologies D) this region shows the level of disruption due to the impact which affecting the molecular, Purkinje, and granule cell layers

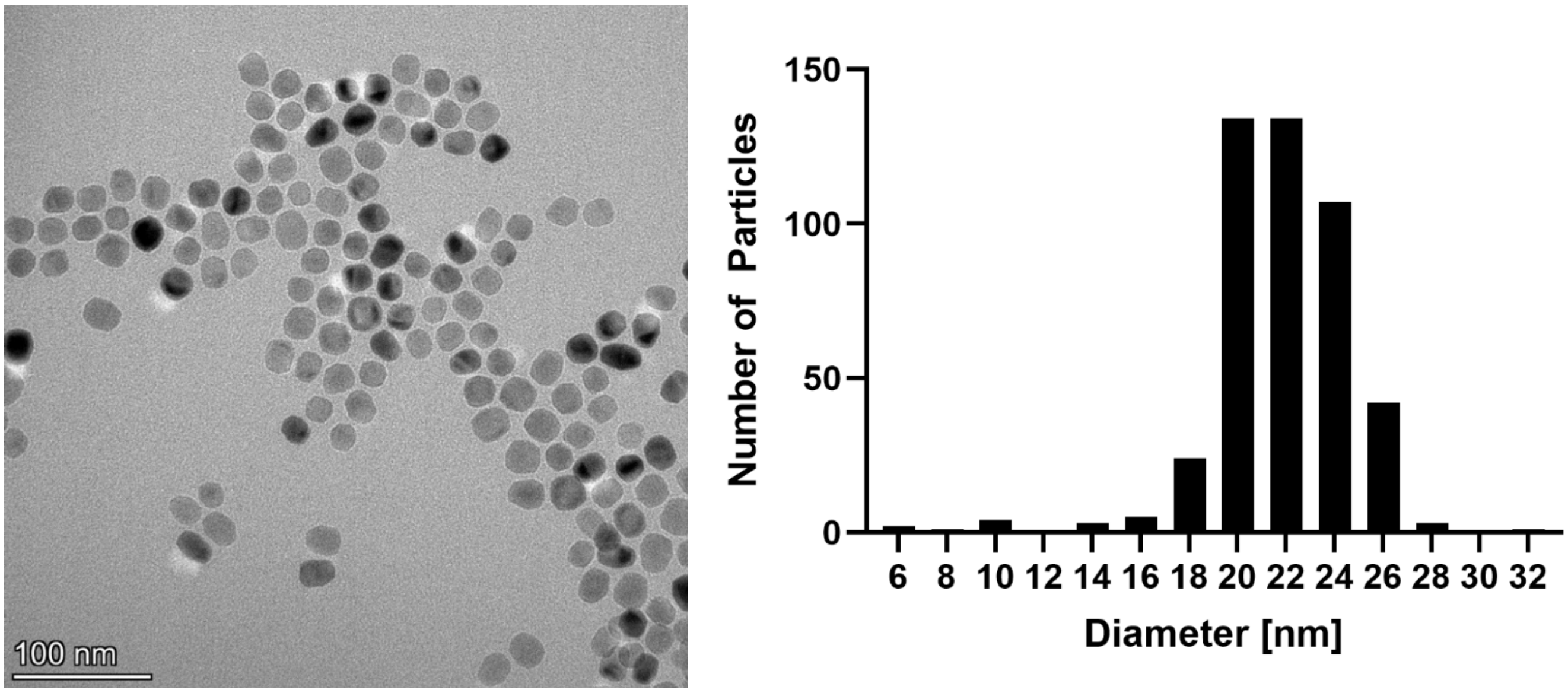


**Supplementary Figure 3: Representative TEM and histogram of the iron oxide nanoparticles.** Transmission electron microscopy (TEM) images were taken of the iron oxide nanoparticles after synthesis. A MATLAB code was used to measure the diameters of the particles, and the results were fitted to a lognormal distribution.

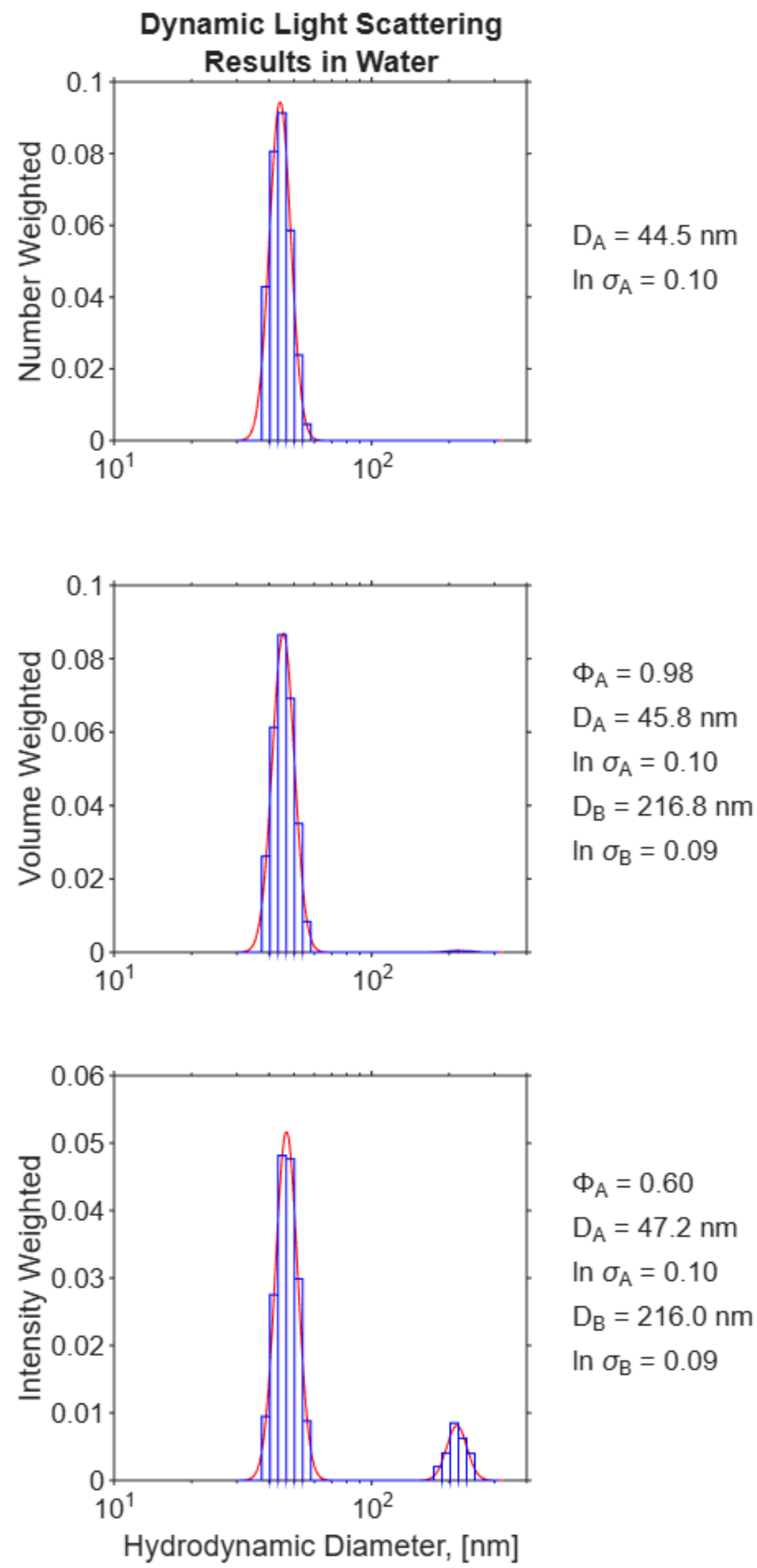


**Supplementary Figure 4: Dynamic Light Scattering (DLS) results for the iron oxide nanoparticles.** DLS measurements were taken of the particles in water to demonstrate colloidal stability. Three representations are presented, as received from the instrument, weighted by intensity, volume, or number distribution. The blue bars are the histogram (logarithmic bins) while the red line are bimodal or unimodal fits. Although the intensity-weighted distribution shows a small peak at ~200 nm, due to the sixth-power dependence of intensity weighted DLS, this likely corresponds to a small number of aggregates. This is further supported by the consistency in the median diameter ($D_A$) and geometric deviation ($\ln(\sigma_A)$) between the number-, volume-, and intensity-weighted distributions.

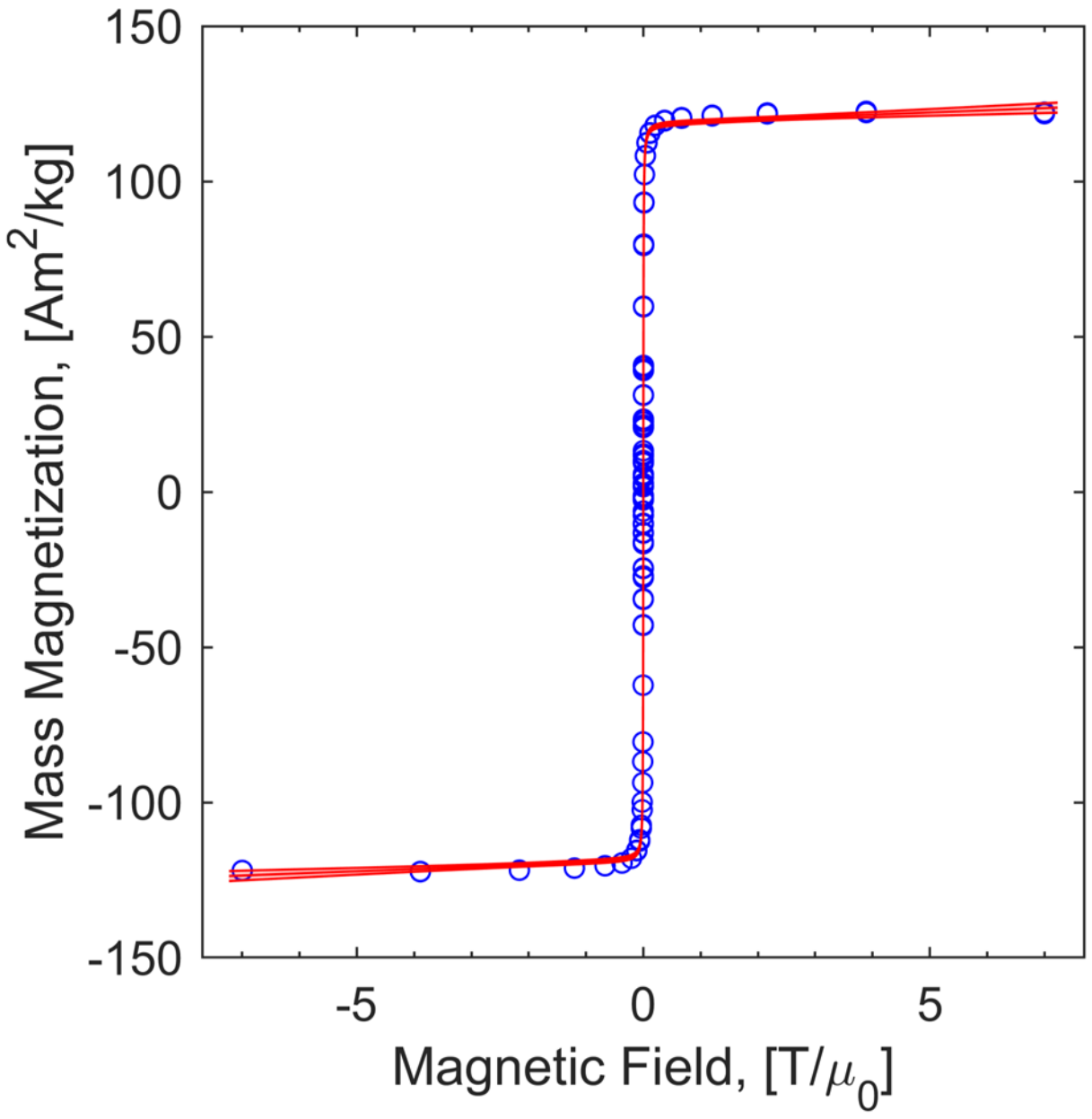


**Supplementary Figure 5: Magnetic characterization of the iron oxide nanoparticles.** Full-range mass magnetization as a function of applied magnetic field strength is shown, with experimental data in blue circles shown alongside a fitted Langevin-Chantrell model as a red line, which had an R-squared value of 0.999.

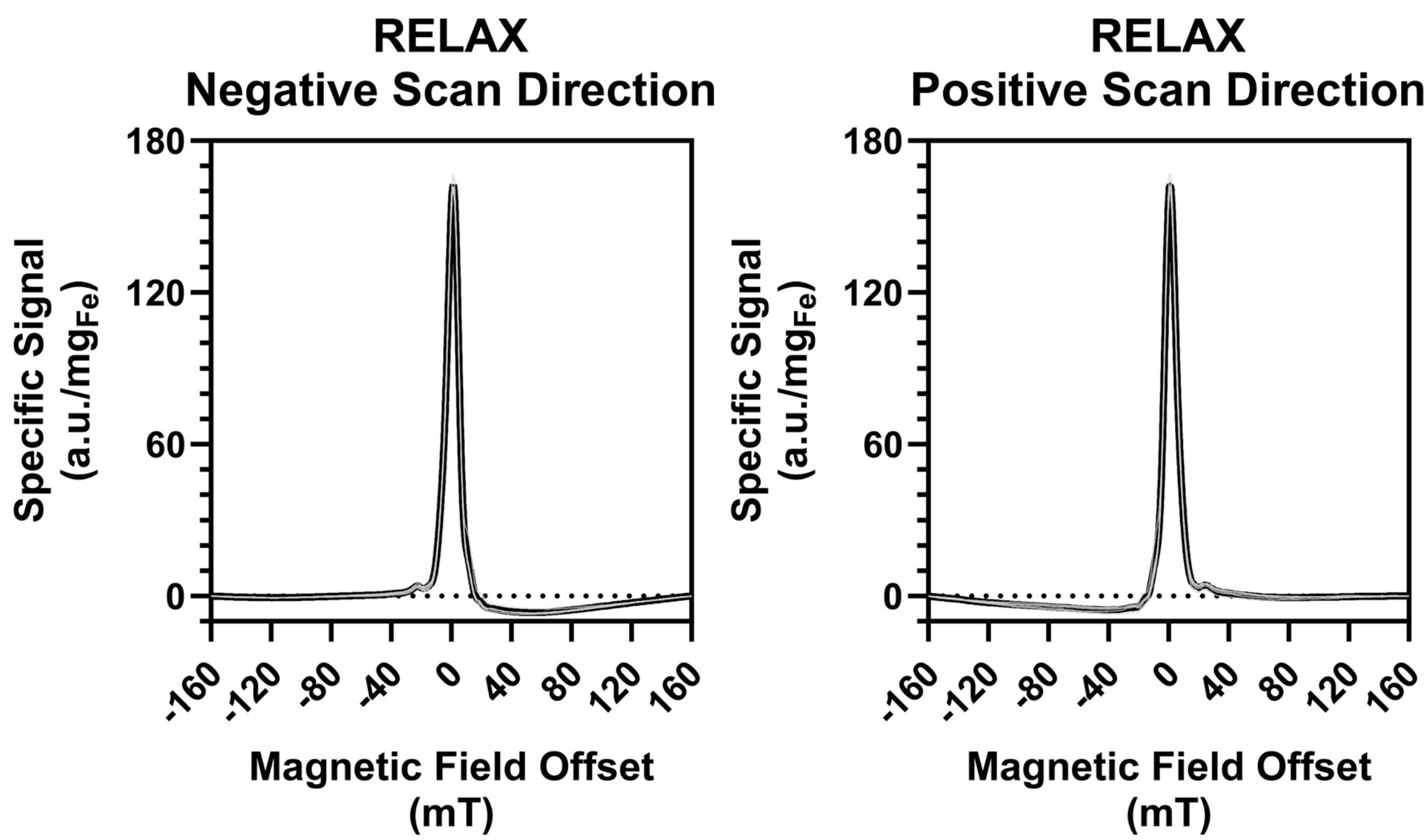


**Supplementary Figure 6: Representative RELAX scans in the MPI for the iron oxide nanoparticles.** RELAX scans were conducted on five technical replicates in both positive and negative direction. The signal is normalized by mass to provide specific signal in arbitrary units per milligram iron. The white lines indicate individual scans while the black lines indicate the average of the five scans.

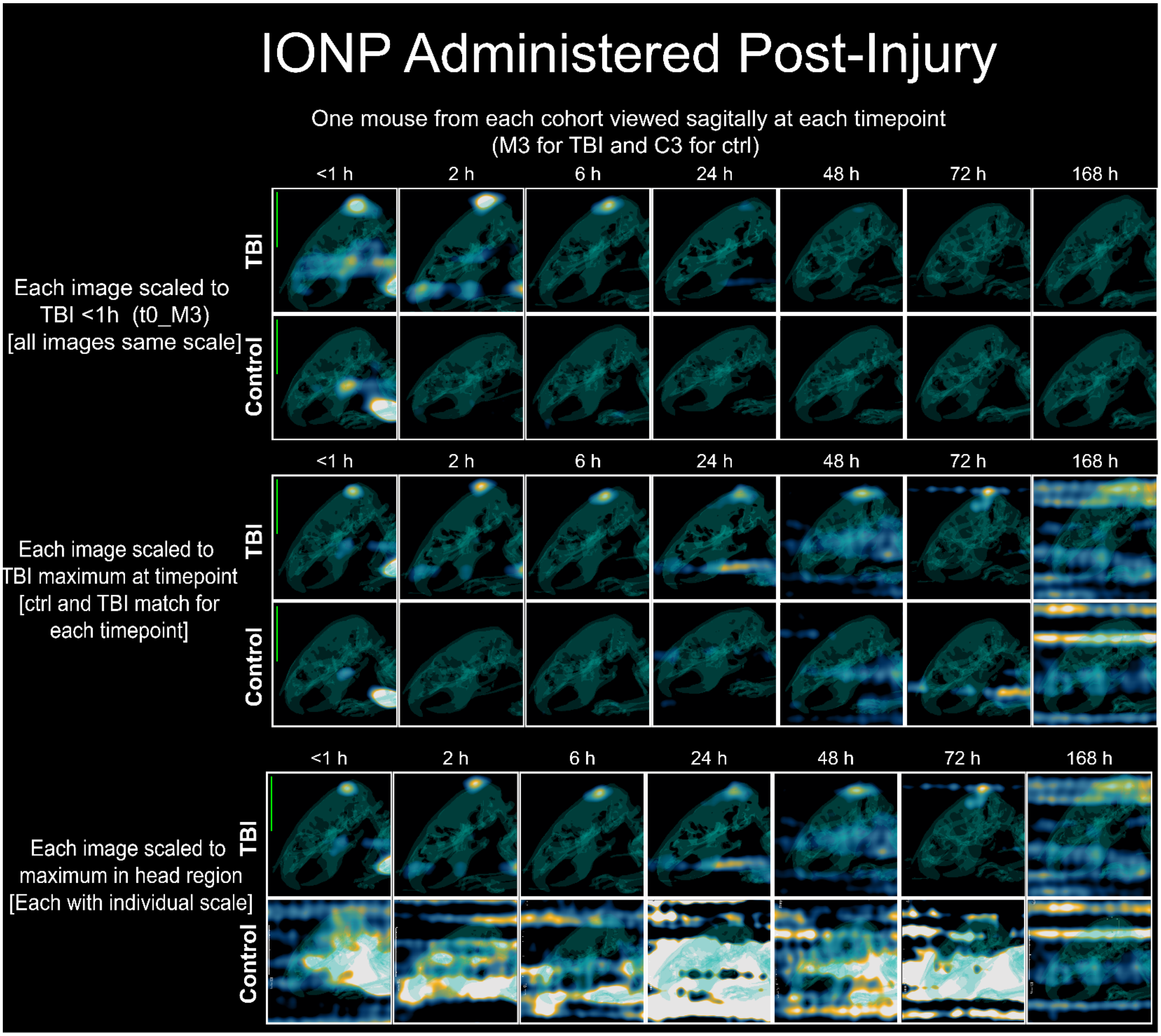


**Supplementary Figure 7: Timelapse for IONP administered post-injury.** MPI/CT timelapse head sagittal images for a representative mouse from each group displayed using different window/leveling strategies. CT volumes are represented as maximum intensity projections and MPI signal is represented as a slice centered at the maximum value in the region of the injury. Top row: For each mouse, the time lapse images have been scaled to the maximum signal in the <1 h image. This view shows how signal decays over time for control and injured mice. Middle row: Each image has been scaled to the maximum signal in the TBI mouse for each time point, facilitating comparison between the control and injury groups over time. This view clearly shows how, even though signal is not apparent in the injury site in the top row for the 24 h and 48 h time points, an MPI signal at the site of injury is evident in the injured mouse when compared to the control mouse. Bottom row: Each image has been scaled to the maximum in the corresponding image. This view shows how signal decays to background noise, albeit with different dynamics in the TBI and control animals.
