# Supplementary material for: Sensitive detection of in vivo traumatic brain injury foci using magnetic particle imaging
Source: Nanoscale. 2026 Sep 24;18(43):23576–89. doi: 10.1039/d6nr02497a (PMC13721992; doi:10.1039/d6nr02497a)
Supplement: Supplementary file 4 [file NR-018-D6NR02497A-s002.pdf]

Supplementary Information (SI) for Nanoscale.
This journal is © The Royal Society of Chemistry 2026

Supporting Information

Supplementary Figure 1 depicts a drawing of the custom in-house-built animal bed parts used for the imaging procedures.

Supplementary Figure 2 depicts IHC images of TBI mice after 7 days of post-impact

Supplementary Figure 3 depicts a representative TEM image of the iron oxide cores after synthesis, where multiple TEMs were used to measure core diameters and distribution of the IONPs.

Supplementary Figure 4 depicts a dynamic light scattering (DLS) histogram and fit of the iron oxide particles after PEG coating.

Supplementary Figure 5 depicts magnetic characterizations of the iron oxide nanoparticles of the iron oxide particles after PEG coating.

Supplementary Figure 6 depicts RELAX scans in the MOMENTUM MPI system, in both the positive and negative directions.

Supplementary Figure 7 depicts a timelapse for representative animals from each group (injured, not injured) in the experimental model where IONP was administered after injury in three different color scaling methods.
